# Combined Experimental and Multivariate Model Approaches for Glycoalkaloid Quantification in Tomatoes

**DOI:** 10.3390/molecules26113068

**Published:** 2021-05-21

**Authors:** Gabriella Tamasi, Alessio Pardini, Riccardo Croce, Marco Consumi, Gemma Leone, Claudia Bonechi, Claudio Rossi, Agnese Magnani

**Affiliations:** 1 Department of Biotechnology Chemistry and Pharmacy, University of Siena, via A. Moro 2, 53100 Siena, Italy; alessio.pardini@unisi.it (A.P.); riccardo.croce@student.unisi.it (R.C.); gemma.leone@unisi.it (G.L.); claudia.bonechi@unisi.it (C.B.); claudio.rossi@unisi.it (C.R.); agnese.magnani@unisi.it (A.M.); 2 Center for Colloid and Surface Science (CSGI), via della Lastruccia 3, 50019 Sesto Fiorentino, Italy; 3 National Interuniversity Consortium of Materials Science and Technology (INSTM), via G. Giusti 9, 50121 Firenze, Italy

**Keywords:** tomatine, chromatography, thermogravimetric analysis, mid-infrared spectroscopy, multivariate analysis

## Abstract

The intake of tomato glycoalkaloids can exert beneficial effects on human health. For this reason, methods for a rapid quantification of these compounds are required. Most of the methods for α-tomatine and dehydrotomatine quantification are based on chromatographic techniques. However, these techniques require complex and time-consuming sample pre-treatments. In this work, HPLC-ESI-QqQ-MS/MS was used as reference method. Subsequently, multiple linear regression (MLR) and partial least squares regression (PLSR) were employed to create two calibration models for the prediction of the tomatine content from thermogravimetric (TGA) and attenuated total reflectance (ATR) infrared spectroscopy (IR) analyses. These two fast techniques were proven to be suitable and effective in alkaloid quantification (R^2^ = 0.998 and 0.840, respectively), achieving low errors (0.11 and 0.27%, respectively) with the reference technique.

## 1. Introduction

Fruit ripening is a complex process that causes considerable changes in color, texture and flavor. Additionally, the chemical composition of fruits is affected during this process, including conversion of starch to sugars, biosynthesis and accumulation of pigments and aromatic volatiles, as well as modification of cell wall ultrastructure [1].

In tomatoes, a significant decrease in steroidal glycoalkaloid content (i.e., α-tomatine and dehydrotomatine) occurs as a function of the ripening process. These components are not useful for plant growth but play a significant role in defense mechanisms against pathogens. According to their specific function, the glycoalkaloid concentration is highest in stems and leaves, during the first stages of plant growth. In the fruits, the glycoalkaloid content decreases as a function of the ripening process and at the same time, the color changes from green to red [2,3,4,5]. It is well known that the tomato glycoalkaloids are bio-synthesized and then degraded during fruit ripening. In particular, in tomato fruits, the tomatine content decreases as the fruits grow and it is completely degraded when the fruits turn red [4,6,7].

It is now well known that glycoalkaloids also possess beneficial effects for human health [6,8,9,10], as well as many other secondary metabolites [11,12,13,14,15,16]. Thus, rapid methods for the quantification of α-tomatine and dehydrotomatine in tomato fruits are potentially useful.

Most of the studies on natural glycoalkaloid quantification are based on chromatographic techniques [17], including the quantification of tomatine [6,7,10]. Although high-performance liquid chromatography (HPLC) still remains the gold standard technique for quantifying organic substances [18], other methods are under development to overcome some disadvantages of this analytical technique. Liquid chromatography is, indeed, very powerful, but it is time-consuming and requires considerable manual work for sample pre-treatments (e.g., extraction and purification of the analytes). In many cases, spectroscopic methods combined with chemometric approaches can overcome these problems. Attenuated total reflection–Fourier transform mid-infrared spectroscopy (ATR-FT-MIR) is widely used because it is a reliable, rapid, low-cost and non-destructive technique. Moreover, combined with chemometric methods, it can highlight the spectral differences between similar samples, modeling the systematic variance of the data and presenting it in a simpler way [19,20,21]. This approach can allow the quantitative determination of bioactive molecules [22]. Although chemometric methods combined with ATR-FT-MIR have been widely applied to classify various food and agricultural products, according to our knowledge, there are no studies about the determination of tomatine in tomato species. Similarly, differential scanning calorimetry (DSC) is a technique that can be applied for quantitative food analysis [23,24]. On the contrary, very few applications of thermogravimetry (TGA) in food analysis are reported, usually related to evaluating thermal stability during processing and the action of environmental conditions more than analyte quantification [25,26]. However, TGA is a very quick and user-friendly technique, and does not require specific sample pre-treatments.

The objective of this study is to evaluate the ability of TGA and ATR-FT-MIR as fast techniques to predict the tomatine content in tomatoes, through multivariate statistical approaches, using HPLC coupled with an electrospray ionization and triple quadrupole mass spectrometer (HPLC-ESI-QqQ-MS/MS) as a comparative reference technique.

## 2. Results and Discussion

### 2.1. HPLC-ESI-QqQ-MS/MS Glycoalkaloid Determination in Different Industrial Tomato Varieties at Different Vine-Ripe Stages

The present study reports the quantification of α-tomatine and dehydrotomatine, in eight industrial varieties of tomatoes, carried out via an HPLC-ESI-QqQ-MS/MS protocol in hydroalcoholic acidic extracts of lyophilized samples. The results are summarized in Table 1. In agreement with other studies [4,6,7,27] on the same variety, a higher content of glycoalkaloids was found in green tomatoes. The turning stage represents the ripening phase at which the most significant changes occur. Indeed, up to a 77% decrease of the two glycoalkaloids was observed. A further decrease between 24 to 77% was observed in the pink ripening stage. Only the H7204 red ripened tomato variety showed a very low α-tomatine content, corresponding to 1.3% of the α-tomatine found in the corresponding green tomatoes, while dehydrotomatine was detected only in trace amounts.

**Figure 1:**
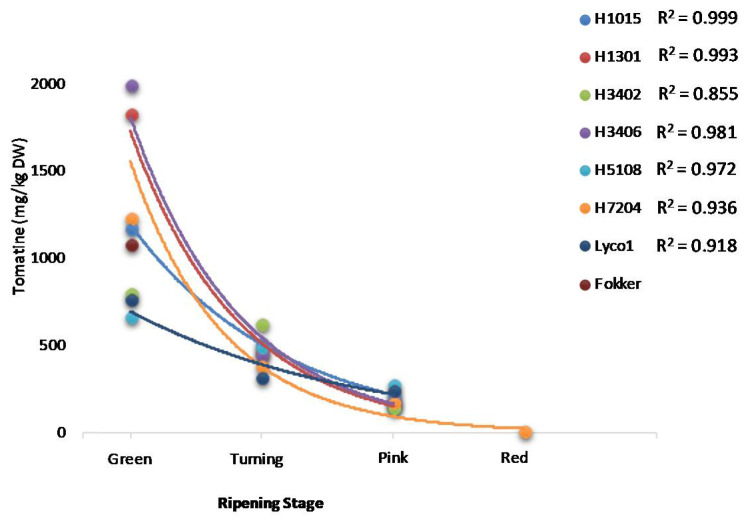
Graphical representation of the pseudo-exponential decrease in tomatine content as a function of the ripening stage for the different industrial tomato varieties. The R^2^ calculated parameters are also reported.

The pseudo-exponential decrease in tomatine (the sum of α-tomatine and dehydrotomatine) as a function of the ripening stage is strictly related to the variety, and the equations that describe the decreasing pattern are reported in Figure 1.

Nevertheless, more significant differences were found among varieties. In particular, H1301 and H3406 varieties were the richest in α-tomatine and dehydrotomatine (Table 1) whereas the lowest values were found in H5108 and Lyco1 varieties. It is possible to notice that the α-tomatine content at the green ripening stage is strongly influenced by the varietal factor, covering a wide range of concentrations of glycoalkaloids (from 1772 ± 33 to 552 ± 45 mg/kg dry weight, DW). The difference in the α-tomatine content among the different tomato varieties became less significant proceeding through the ripening stages, from the green to the pink ripening stage. In the latter, the range of the α-tomatine content was rather narrow, ranging from 238 ± 17 to 120 ± 11 mg/kg DW. Interestingly, the rate of α-tomatine degradation, due to the ripening process of the fruits, also differed within the different varieties, underlining that this process is mainly influenced by the intrinsic genetic differences linked to the variety of the plants.

### 2.2. Thermogravimetric Analysis (TGA)

Thermogravimetry has already been applied to analyze and quantify vegetal compounds in complex matrices [28,29]. The weight loss of each freeze-dried sample in the ranges 120–200 and 200–400 °C are summarized in Table 2.

The weight loss in the range 120–200 °C increases from 16 ± 1% for tomatoes at the green stage to 18.7 ± 0.5% and 21 ± 1% for tomatoes at the turning and pink stages, respectively. Concerning the decomposition between 200–400 °C, a clear trend can be observed in the analyzed samples: moving from the green to pink stage, a decrease in weight loss is observed. Indeed, in the green stage, a mean weight loss of about 38 ± 1% is found that decreases to 36 ± 1% in the turning stage and reaches 33.7 ± 0.4% in the pink stage.

**Figure 2:**
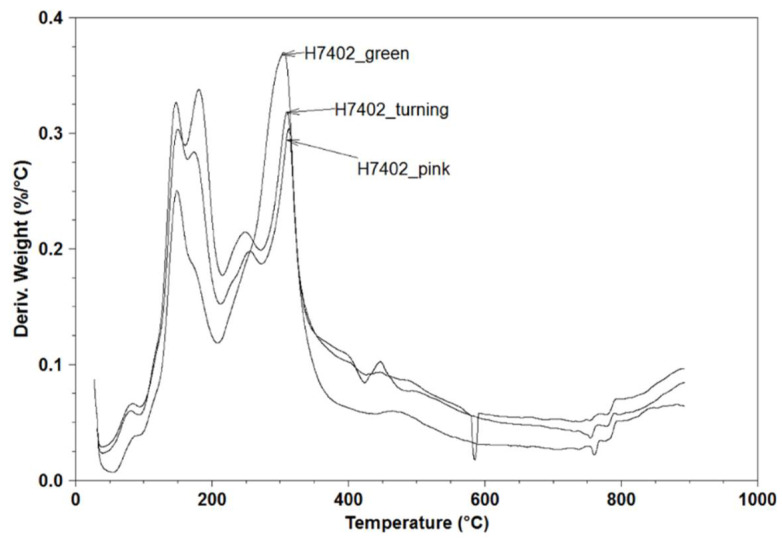
Thermographs of H7204 at different ripening stages.

In Figure 2, a comparison between thermographs of H7204 variety samples at different ripening stages is depicted, showing the peculiar increase in weight loss between 120 and 200 °C. In this temperature range, the degradation of volatiles occurs [30]. The increase in weight loss observed in this region of the thermogram could be explained by the accumulation of flavor and aromatic compounds as a consequence of the ripening process. Concurrently, a decrease in weight loss between 200 and 400 °C is observed. At these temperatures, macromolecules such as cellulose, hemicellulose, pectin and starch are thermally degraded [30,31]. During the ripening process, many hydrolytic enzymes are involved in cell wall metabolism, causing the softening of fleshy fruits. At the same time, the depolymerization of many polysaccharides to sugars occurs. Consequently, the content of these macromolecules in tomatoes progressively decreases as a function of the ripening process.

**Figure 3:**
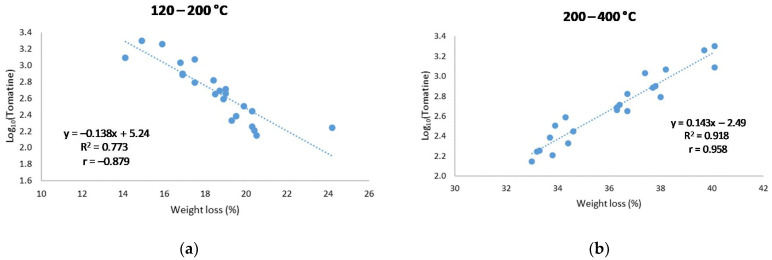
Linear regression model of the Log_10_(Tomatine) vs. the weight loss in the temperature ranges (**a**) 120–200 °C and (**b**) 200–400 °C.

As shown in Figure 3, strong correlations showing opposite trends were found between the logarithm of the tomatine content detected by HPLC-ESI-QqQ-MS/MS and the weight loss in both of the temperature ranges (Pearson’s correlation coefficients, r = −0.879 and 0.958, respectively).

**Figure 4:**
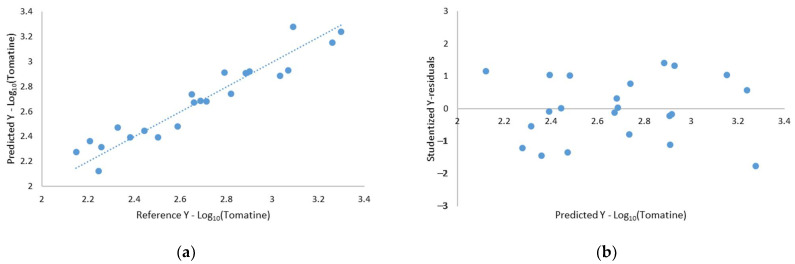
(**a**) MLR output of measured vs. predicted values of tomatine concentration and (**b**) model residuals.

Therefore, a multiple linear regression (MLR) model was proposed to extrapolate the tomatine concentration in lyophilized tomato samples from TGA, taking into account the weight loss in the selected temperature ranges. The MLR analysis gave the equation Y = −0.0472X_1_ + 0.0983X_2_ (R^2^ = 0.999 and adjusted R^2^ = 0.998). The intercept of the regression model was forced to zero since it was not statistically significant (*p* > 0.05), while both the coefficients of X_1_ and X_2_ were significantly different from zero (*p* << 0.05). The F-test showed that the overall model is significant (*p* << 0.05), with a root mean square error (RMSE) of 0.11. The relationship between the tomatine concentrations predicted by the MLR model and the measured ones is reported in Figure 4a. The residual plot of the model (Figure 4b), reporting the autoscaled Y-residuals vs. the predicted Y, gave a random distribution between ± 3 (99% confidence interval) for all the samples, confirming that the MLR model reported here is able to predict tomatine values along the whole range of concentrations employed.

### 2.3. Attenuated Total Reflection–Fourier Transform Mid-Infrared Spectroscopy (ATR-FT-MIR)

**Figure 5:**
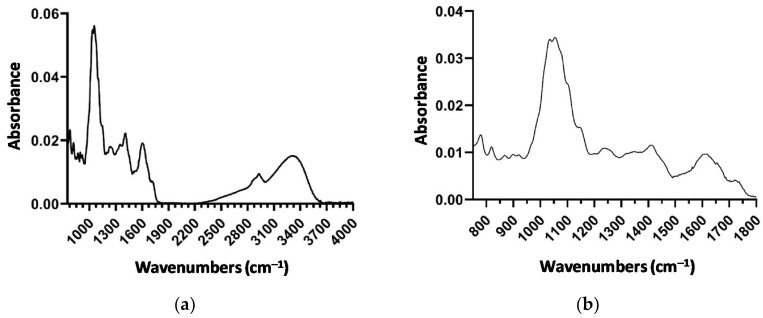
(**a**) Example of ATR-FT-MIR spectrum of lyophilized tomato. (**b**) Average ATR-FT-MIR spectrum of 22 samples in the range 750–1800 cm^−1^.

Figure 5a reports the spectrum of a dried tomato sample (as an example). None of the spectra deviate from the example, if not considered in terms of peak intensity. In the spectra displayed, the specific bands of vegetal samples are highlighted and assigned as follows. The band at 3289 cm^−1^ is characteristic of NH and OH stretching vibrations. The region of 2923–2853 cm^−^^1^ can be assigned to the symmetrical and asymmetric stretching modes of the CH_3_ and CH_2_ groups. The 1720 cm^−^^1^ band corresponds to the stretching of the C=O ester carbonyl or carboxylic acid groups, which are characteristic of fatty acids and polysaccharides. The amide I band at 1652 cm^−1^ results from the C=O stretching in the amides I, II and III, while the amidic band II at 1520 cm^−1^ originates from the bending vibrations of the N-H groups.

Table 3 summarizes bands in the IR region from 1800 to 900 cm^−1^ that include the “fingerprint” region, which includes bands corresponding to the vibrations of the C-O, C-C, C-H and C-N bonds. This region is, on the one hand, very rich in information, but, on the other hand, difficult to analyze due to its complexity. This area provides important information about organic compounds, such as sugars, alcohols and organic acids, present in the sample by featuring their molecular vibrations (stretching, bending and torsions of the chemical bonds) in specific infrared regions. This region was dominated by a broad band centred at 1055, 1035 cm^−1^, with evident shoulders at 1145 and 1100 cm^−1^, due to strong vibrational modes of various carbohydrates and acids, which are abundant groups in tomatoes. Tomatine shows main bands in the region between 1100 and 950 cm^−1^, which, however, are covered by the stronger sugar and polysaccharide absorption and are not useful for quantitative analysis as the absorbance band at 956 cm^−1^ corresponds to a *trans* –CH=HC– bending out of plane deformation band that is the unique IR marker band specific to lycopene [32].

As discussed, the spectra contain a multitude of bands that are characteristic of vegetal samples which do not allow to obtain information on a specific compound without the interference of the matrix as whole. For this reason, we used an ATR-FT-MIR “fingerprint” analytical approach [21,34] for the structural identification of compounds considering that no two chemical structures will have the same ATR-FT-MIR spectrum. ATR-FT-MIR provides a characteristic signature of chemical or biochemical substances present that can be used for chemometric studies.

### 2.4. Chemometric Approach

The average spectrum of 22 samples, as regards the most informative regions located at low wavenumbers (752–1800 cm^−^^1^), is reported in Figure 5b. This was used to build the regression model. The predictive model for the determination of tomatine in extracted tomato samples was obtained by applying the partial least squares regression (PLSR) model to 16 samples of the calibration set, after data pre-treatment and column mean centering. The uncertainty test identifies 48 significant variables, leading to a decrease in the sample/variable ratio. This also determines a decrease in the risk of finding random correlation and leads to an increase in the reliability of the regression model [35].

The first two components have been identified as significant for tomatine quantification. In more detail, in the final model, the first three latent variables explain 95% of the Y-variance and 73% of the variance in the X-block, with Pearson’s regression coefficient R^2^ = 0.95 and root mean square of calibration (RMSEC) = 0.11.

**Figure 6:**
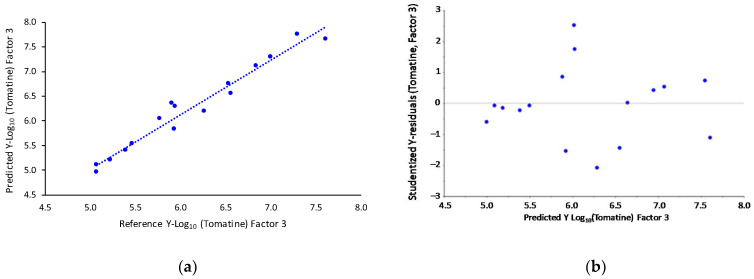
PLS output, (**a**) measured vs. predicted values of tomatine concentration and **(b**) model residuals.

Figure 6a shows the relationship between the measured values and the predicted ones for the calibration set; all the samples are randomly distributed around the regression line with a negligible dispersion for the whole range of variability. In Figure 6b, the relationship between the autoscaled Y-residual and the predicted ones is shown: all the samples are randomly distributed within ± 3 values that correspond to the 99% confidence interval. Therefore, it is possible to confirm that the regression model presents a comparable predictability along the whole concentration interval with a negligible BIAS value.

Finally, the model was validated on the test set. The tomatine contents, predicted by the PLS regression model, were compared with the experimental data to evaluate the actual predictability of the proposed model. The performances were evaluated through the joint analysis of the Pearson’s regression coefficient, R^2^_pred_, and the root mean square error in prediction, RMSEP:

(1) $$\mathrm{RMSEP}=\sqrt{\frac{\sum_{i}^{N}{\left(y_{i}-{\hat{y}}_{i}\right)}^{2}}{N}}$$

where $y_{i}-{\hat{y}}_{i}$ is the difference between the measured and predicted value for the i-th sample in the test set. 

The quality parameters calculated for the proposed model confirmed its goodness in predicting tomatine concentration, from spectral features of tomato extract, showing a high R^2^_pred_ in prediction (0.84) and a low RMSEP (0.27), as well as a negligible BIAS value (0.08). 

Table 4 reports the comparison between experimental data obtained via HPLC-ESI-QqQ-MS/MS determination of tomantine and predicted data through the combined experimental/statistic approaches: TGA/MLR and the ATR-FT-MIR/PLS model. The data have been statistically compared via two-way ANOVA followed by Dunnett’s test. It is evident that the results are not statistically different considering the 95% of confidence interval (Dunnett’s test, *p* >> 0.05). This result underlines that the two alternative semi-quantitative methods, TGA/MLR and ATR-FT-MIR/PLS, that do not require any pre-treatment extraction of the lyophilized and ground tomato material, are reasonable alternatives to the quantitative HPLC-ESI-QqQ-MS/MS determination (strictly dependent on a solid/liquid extraction).

In conclusion, the complex changes occurring during the ripening process produce significant changes in the chemical composition of tomato fruits. TGA and ATR-FT-MIR analyses revealed significant differences in tomatoes at the different ripening stages, probably due to the variation in the macromolecule content, as a consequence of the enzymatic reactions occurring during fruit growth and maturation. Through chemiometric approaches, these variations were found to correlate with the glycoalkaloid content. The MLR and the PLS regression models constructed on the values detected by HPLC-ESI-QqQ-MS/MS analyses as a reference allowed us to accurately quantify tomatine in the tomato samples via TGA and ATR-FT-MIR analyses. These two techniques may represent a valid alternative in the quantification of tomatine in tomatoes, permitting the omission of the pre-treatments required for chromatographic analyses.

## 3. Materials and Methods

### 3.1. Chemicals

All the following solvents were purchased from Sigma-Aldrich (Milan, Italy): ethanol (EtOH, gradient grade ≥ 99.9%) and methanol (MeOH, gradient grade ≥ 99.9%), glacial acetic acid (CH_3_COOH, reagent grade ≥ 99.8%) and formic acid (HCOOH, ACS grade ≥ 98%). Tomatidine hydrochloride (≥95%) was also purchased from Sigma-Aldrich whereas tomatine (>75%) was purchased from TCI Europe (Belgium). Ultra-pure deionized water was produced using an Acquinity P/7 purifier system (MembraPure GmbH, Berlin, Germany).

### 3.2. Plant Materials

**Figure 7:**
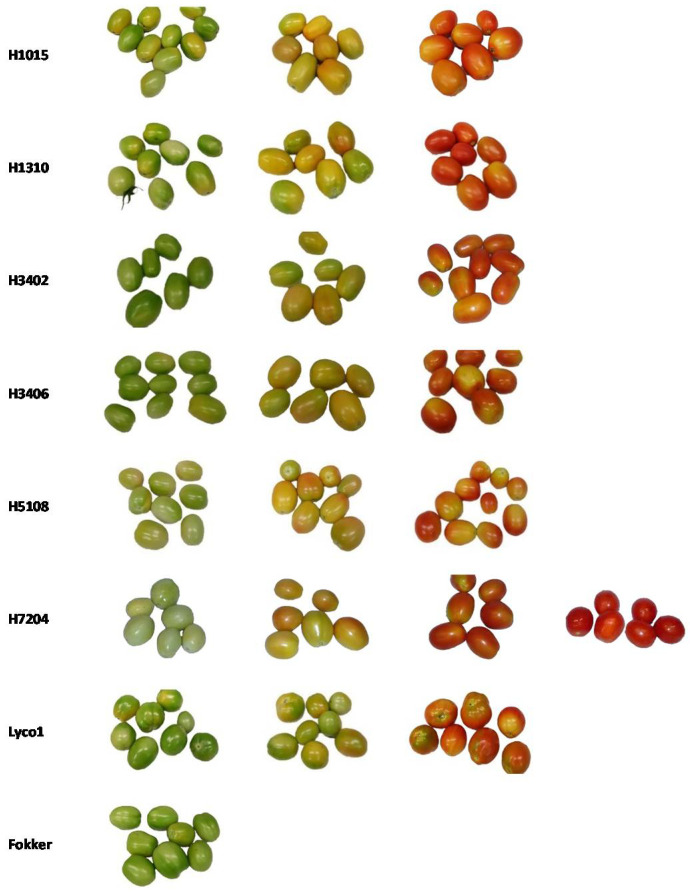
Industrial tomato varieties at different vine-ripening stages (green, turning, pink and red), classified according to the California Tomato Commission and United States Department of Agriculture (USDA) guidelines.

Eight different vine-ripened industrial varieties of tomato fruits, harvested in summer 2017 at different ripening stages (green, turning, pink and red) were analyzed (Figure 7). The samples were classified according to the definitions provided by the California Tomato Commission and United States Department of Agriculture (USDA, [36]): green (the surface of the tomato is completely green in color, the shade of green may vary from light to dark), turning (more than 10%, but not more than 30%, of the total surface shows a definite change in color from green to tannish-yellow, pink, red or a combination thereof), pink (more than 30%, but not more than 60%, of the total surface is pink or red in color). 

The samples were washed with deionized water and dried. Six tomatoes of each variety at the different ripening stages were homogenized with a blender (Moulinex, 1000 W) and the homogenates were freeze-dried (VirTis BenchTop Pro lyophilizer, −51 ± 2 °C, 1.3 ± 0.5 mbar) until a constant weight (5 days, averaged samples water content, 92 ± 1%). Lyophilized samples were then ground in porcelain mortar, sieved to a 500 µm particle size and stored at −20 ± 1 °C before the subsequent analyses.

### 3.3. HPLC-ESI-QqQ-MS/MS Quantification of Glycoalkaloids

Glycoalkaloid extraction and analytical quantification were carried out via HPLC-ESI-QqQ-MS/MS as previously validated and reported [6,7], with slight modifications. Briefly, lyophilized samples were extracted by an hydroalcoholic acidic mixture consisting of EtOH/CH_3_COOH 1%, (70:30, *v*/*v*) and the extraction was ultrasound assisted (two-cycle extraction on the solid phase). The extracts were dried under nitrogen flow with subsequent lyophilization and then reconstituted in MeOH 80%. The analytical determination of α-tomatine and dehydrotomatine was performed by HPLC-ESI-QqQ-MS/MS analyses. Each sample was extracted in triplicate. The separation was carried out by a reverse-phase column (Phenomenex Luna C18(2)-HST, 2.5 µm, 100 × 2.0 mm, 5 μm, 100 Å equipped with a Phenomenex SecurityGuard ULTRA pre-column) thermostatted at 30 ± 1 °C and a linear gradient with (A) H_2_O and (B) MeOH both acidified with formic acid 0.1% (*v*/*v*): linear gradient 40–90% B in 15 min (flow rate of 0.2 mL/min). The injection volume was 5 μL. The ESI-MS conditions were optimized through the direct injection of a tomatine standard solution in MeOH in positive ionization mode and α-tomatine and dehydrotomatine were quantified by multiple reaction monitoring mode (MRM; collision energy −31 V and scan time, 500 ms). The analytical quantification was carried out via an external calibration method: linearity range, 0.5–30 mg/L of tomatine (a mixture of α-tomatine, 85% and dehydrotomatine, 13%). The tomatidine was used as an internal standard, taking into account that it does not naturally occur as aglycone in tomatoes [4,6,7]. Calibration curves showing equations with R^2^ > 0.990 were used for the quantification. The limit of detection (LOD) and limit of quantification (LOQ) were: 0.20//0.05 and 0.50//0.20 mg/L for α-tomatine and dehydrotomatine, respectively. The results were expressed as mg/kg of sample dry weight (DW).

All the samples were extracted in triplicate and each extract was analyzed three times (*n* = 9). and results were reported as mean ± standard deviation (SD). The significant differences between means were assessed by analysis of variance (ANOVA) followed by Tukey’s post hoc test. All the statistical treatments were run on Microsoft Office Excel 365, implemented with the Real Statistic subroutine, setting the level of significance at *p* < 0.05.

### 3.4. Thermogravimetric Analysis (TGA)

The analyses were performed using a Q1000 thermogravimeter (TA, Instruments) applying a thermal program from 30 to 900 °C with a heating ramp of 10 °C/min, under constant nitrogen flow [37]. Three lyophilized aliquots for each variety (15 mg in weight) were analyzed.

### 3.5. Attenuated Total Reflection–Fourier Transform Mid-Infrared Spectroscopy (ATR-FT-MIR)

All the samples were analyzed by FTIR using a Nicolet IS50 FTIR spectrophotometer (Thermo Nicolet Corp., Madison, WI, USA), equipped with a single-reflection germanium ATR crystal (Pike 16154, Pike Technologies, Madison, WI, USA) and a deuterated triglycine sulphate (DTGS) detector. The spectra were acquired (32 scans per sample or background) in the range of 4000–800 cm^−1^ at a nominal resolution of 4 cm^−1^. The spectra were corrected using the background spectrum of air. The analysis was carried out at room temperature by spreading a lyophilized sample onto the surface of the ATR crystal. Before each sample was analyzed, the ATR crystal was carefully cleaned with water-wet cellulose tissue and dried using a flow of pure nitrogen gas. The cleaned crystal was checked spectrally to ensure that no residue was retained from the previous sample. The spectrum of every sample was collected 3 times to check the reproducibility and do a statistical analysis. It should be noted that the individual spectra of the same tomato did not vary in band wavelength and varied to some extent in the absorbance. However, the characteristic signatures remained very similar, so averaged spectra are shown in the results.

The frequency scale was internally calibrated with a helium–neon reference laser to an accuracy of 0.01 cm^−1^. OMNIC software (OMNIC software system Version 9.8 Thermo Nicolet) was used for spectra acquisition and manipulation [38].

### 3.6. Multiple Linear Regression (MLR)

The multiple linear regression (MLR) analysis was performed in Microsoft Office Excel 365, implemented with the Real Statistic subroutine, plotting tomatine concentration (Y) determined by HPLC-ESI-QqQ-MS/MS analyses vs. the weight loss detected by TGA analyses in the range of temperature, 120–200 °C (X_1_) and 200–400 °C (X_2_). Since the concentration of tomatine ranged over one order of magnitude, the values were converted in the logarithmic form to make the data distribution symmetrical. To build the MLR model, the H7204 sample at the red ripening stage was discarded as a leverage outlier, since its tomatine content was too low. In this way, the distribution of tomatine content was fairly symmetrical, allowing us to build a reliable regression model.

### 3.7. Chemometric Approach

#### 3.7.1. Data Processing

The ATR-FT-MIR spectra were imported into UnscramblerX Software (Camo Analytics, Oslo, Norway) for multivariate data analysis. ATR-FT-MIR spectra absorbances at different wavelengths were stored in a data matrix with samples placed in rows and reference values of tomatine placed in the first column. As extensively reported in the literature [39,40,41,42], it is necessary to split the entire database into two subsets, to evaluate the performance of the prediction model. The first subset, representing 75% of the available samples, was used to build the regression model, whilst the second subset, consisting in 25% of total samples, was used to estimate the actual predictability of the model. Therefore, the dataset was divided into a calibration set and a test set using random sample selection in order to avoid a biased model. In addition, an internal cross-validation step was performed using the venetian blind algorithm to find the best modeling settings (i.e., the number of latent variables, [43]). This procedure consisted in building, optimizing and testing models to obtain reliable prediction of tomatine concentration for future extracted tomato samples.

#### 3.7.2. Spectral Pre-Processing

The ATR-FT-MIR spectra of the 22 samples were averaged and the most informative region was located at low wavenumbers and the regression model was built using the spectral features from 752 cm^−1^ to 1800 cm^−1^. The H7204 sample at the red ripening stage was also discarded in this case. 

Usually, when a regression model is built from spectral data, it is necessary to pre-treat the input variables to remove useless information resulting from unwanted systematic variations. In the present work, a combination of standard normal variate (SNV) and second derivative were used to pre-treat spectral data, removing the baseline offset and differences in global signal intensity [44,45,46]. SNV transforms the original data according to the following equation:

(2) $${\left(x_{\mathrm{ij}}\right)}_{\mathrm{SNV}}=\frac{x_{\mathrm{ij}}-{\;\bar{x}}_{i}}{s_{i}}$$

where $x_{\mathrm{ij}}$ represents the absorbance for the j-th wavelength and the i-th sample, ${\;\bar{x}}_{i}$ and $s_{i}$ are the average and the standard deviation of the wavelengths for the i-th spectrum, respectively. For this reason, SNV transformation is also known as row autoscaling.

A second derivative transformation was also applied to FT-MIR spectra pre-treated with standard normal variate (SNV) transformation. In more detail, a Savitszi–Golay algorithm was applied using a second order polynomial and symmetric kernel option with 7 smoothing points [46].

#### 3.7.3. Partial Least Squares Regression (PLSR) and Martens’ Uncertainty Test

Partial least squares regression (PLSR) was used on the calibration set to obtain the model that was subsequently used to predict tomatine content in industrial tomato samples. Additionally, in this case, tomatine concentration values were converted into the logarithmic form to make the distribution fairly symmetrical. 

A kernel PLS algorithm was used to correlate the logarithm of tomatine concentration and spectral features transformed as mentioned previously [47]. The best number of latent variables that was used in the regression function was estimated for the cross-validation procedure, taking into account the cross-validation root mean square error (RMSECV) trend with respect to the number of retained components. In the present work, only three components were necessary to explain the majority of the total X- and Y-variance. Once the optimal number of latent variables were chosen, Martens’ uncertainty test [48] was performed in order to remove unimportant information within spectral data. This powerful tool allowed us to improve the predictability of the model, retaining only the significant variable by giving a more reliable estimate of the prediction error when the model was tested on new samples. Moreover, since a reduced number of spectral variables were used, a simpler model was generated.

In Martens’ uncertainty test, the regression coefficients Bi for each cross-validation sub-model, chosen with the venetian blind option, were calculated and the differences from the regression coefficient of the total model, B_tot_, were computed. The sum of the squares of the differences in all sub-models was finally evaluated in order to obtain an expression of the variance of the Bi estimate for a specific wavelength. With a *t*-test, the significance (confidence level of 95%) of the estimate of Bi was calculated and the resulting regression coefficients were presented with uncertainty limits. Variables with uncertainty limits that did not contain the zero were significant variables. This procedure was iteratively repeated until the difference between RMSEC and RMSECV reached a minimum value.

## Figures and Tables

**Table 1:** α-Tomatine and dehydrotomatine contents in vine-ripened industrial tomato varieties. The values are expressed as mg/kg DW and mean ± SD (standard deviation; *n* = 9). The content of tomatine (sum of the two glycoalkaloids) is also reported.

Variety	Ripening Stage	α-Tomatine	Dehydrotomatine	Tomatine
H1015	Green	1028 ± 53 ^a^	147 ± 9 ^a^	1176 ± 54 ^a^
	Turning	441 ± 28 ^b^	78 ± 8 ^b,c^	519 ± 29 ^b^
	Pink	180 ± 19 ^c,d,e^	34 ± 5 ^d,e^	213 ± 20 ^c,d^
H1301	Green	1614 ± 40 ^f^	215 ± 4 ^f^	1829 ± 41 ^e^
	Turning	400 ± 17 ^b^	57 ± 3 ^g^	457 ± 17 ^b,f^
	Pink	141 ± 12 ^c^	21 ± 2 ^h^	161 ± 12 ^c,g^
H3402	Green	688 ± 40 ^g^	109 ± 11 ^i^	796 ± 41 ^h^
	Turning	535 ± 19 ^h^	86 ± 7 ^c,j^	621 ± 20 ^i^
	Pink	120 ± 11 ^c^	21 ± 3 ^h^	141 ± 11 ^g^
H3406	Green	1772 ± 33 ^i^	221 ± 3 ^f^	1993 ± 33 ^j^
	Turning	388 ± 18 ^b^	61 ± 4 ^g^	449 ± 18 ^f,k^
	Pink	155 ± 11 ^c,d^	26 ± 3 ^d,h^	181 ± 12 ^c,d,g^
H5108	Green	552 ± 45 ^h^	111 ± 7 ^i^	663 ± 46 ^i^
	Turning	420 ± 27 ^b^	69 ± 7 ^b^	489 ± 28 ^b,f^
	Pink	238 ± 17 ^e,j^	42 ± 6 ^e^	279 ± 18 ^l,m^
H7204	Green	1088 ± 34 ^a^	143 ± 12 ^a^	1231 ± 36 ^a^
	Turning	314 ± 16 ^k^	75 ± 6 ^b,c^	388 ± 17 ^k^
	Pink	148 ± 17 ^c^	28 ± 4 ^d,h^	176 ± 17 ^c,g^
	Red	14 ± 2 ^l^	<LOD	14 ± 2 ^n^
Lyco1	Green	678 ± 62 ^g^	90 ± 8 ^j^	768 ± 63 ^h^
	Turning	294 ± 22 ^j,k^	26 ± 3 ^d,h^	320 ±22 ^m^
	Pink	217 ± 32 ^d,e^	25 ± 4 ^d,h^	242 ± 32 ^d,l^
Fokker	Green	952 ± 101 ^m^	128 ± 15 ^k^	1080 ± 102 ^o^

Values marked with the same letter within the same column are not statistically different (Tukey’s test, *p* > 0.05).

**Table 2:** Results of TGA analysis: mean ± SD (*n* = 3) of the weight loss (in percentage) in the ranges 120–200 °C and 200–400 °C as a function of ripening stages.

Variety	Ripening Stage	Weight Loss	
		120–200 °C	200–400 °C
H1015	Green	17.5 ± 0.2	38.2 ± 0.3
	Turning	19.0 ± 0.2	36.4 ± 0.2
	Pink	19.3 ± 0.2	34.4 ± 0.5
H1301	Green	15.9 ± 0.1	39.7 ± 0.8
	Turning	19.0 ± 0.4	36.3 ± 0.3
	Pink	20.4 ± 0.3	33.8 ± 0.2
H3402	Green	16.9 ± 0.3	37.8 ± 0.4
	Turning	17.5 ± 0.5	38 ± 0.1
	Pink	20.5 ± 0.1	33 ± 0.3
H3406	Green	14.9 ± 0.1	40.1 ± 0.2
	Turning	18.5 ± 0.6	36.7 ± 0.3
	Pink	20.3 ± 0.2	33.3 ± 0.5
H5108	Green	18.4 ± 0.3	36.7 ± 0.6
	Turning	18.7 ± 0.2	36.3 ± 0.5
	Pink	20.3 ± 0.4	34.6 ± 0.2
H7204	Green	14.1 ± 0.2	40.1 ± 0.3
	Turning	18.9 ± 0.3	34.3 ± 0.4
	Pink	24.2 ± 0.3	33.2 ± 0.6
Lyco1	Green	16.9 ± 0.5	37.7 ± 0.4
	Turning	19.9 ± 0.4	33.9 ± 0.6
	Pink	19.5 ± 0.2	33.7 ± 0.4
Fokker	Green	16.8 ± 0.2	37.4 ± 0.5

**Table 3:** Main functional groups assigned to ATR-FTIR spectra of tomato [33].

Wavenumber (cm^−1^)	Proposed Assignment
1720	C=O ester
1650	Amide I, *β*-sheet
1604	C–C aromatic
1551	C–C aromatic
1520	Amide II, C≡N stretching
1410	CH_2_ bending of lipids and fatty acids
1350	CH_3_ bending proteins and lipids and CH_2_ wagging and twisting
1240	OH bending
1196	C–O–C ester stretching
1145	C–O–C ester stretching
1055	C–O–C glycosidic bond
955	CH(trans OOP)

**Table 4:** Comparison between HPLC-ESI-QqQ-MS/MS experimental data and TGA and ATR-FT-MIR predicted data, via MLR and PLS statistic models, respectively. The values within the same row are not statistically different (two-way ANOVA, Dunnett’s test, *p* >> 0.05).

Variety	Ripening Stage	Tomatine		
		HPLC-ESI-QqQ-MS/MS Experimental	TGA/MLR Model Predicted	ATR-FT-MIR/PLS Model Predicted
H1015	Green	1176 ± 54	851	1177
	Turning	519 ± 29	481	482
	Pink	213 ± 20	296	213
H1301	Green	1829 ± 41	1422	1820
	Turning	457 ± 17	470	458
	Pink	161 ± 12	229	160
H3402	Green	796 ± 41	830	797
	Turning	621 ± 20	813	621
	Pink	141 ± 11	189	144
H3406	Green	1993 ± 33	1735	1874
	Turning	449 ± 18	544	454
	Pink	181 ± 12	207	182
H5108	Green	663 ± 46	550	664
	Turning	489 ± 28	486	461
	Pink	279 ± 18	278	280
H7204	Green	1231 ± 36	1893	1182
	Turning	388 ± 17	302	371
	Pink	176 ± 17	133	177
Lyco1	Green	768 ± 63	811	742
	Turning	320 ±22	248	325
	Pink	242 ± 32	247	239
Fokker	Green	1080 ± 102	766	1039

## Data Availability

Data sharing not applicable.

## Sample Availability

Samples are not available.

## References

[1] Giovannoni J. (2001). Molecular biology of fruit maturation and ripening. Annu. Rev. Plant. Biol..

[2] Friedman M. (2002). Tomato glycoalkaloids: Role in the plant and in the diet. J. Agric. Food Chem..

[3] Friedman M., Levin C.E., Lee S.U., Kim H.J., Lee I.S., Byun J.O., Kozukue N. (2009). Tomatine-containing green tomato extracts inhibit growth of human breast, colon, liver, and stomach cancer cells. J. Agric. Food Chem..

[4] Friedman M. (2013). Anticarcinogenic, cardioprotective, and other health benefits of tomato compounds lycopene, α-tomatine, and tomatidine in pure form and in fresh and processed tomatoes. J. Agric. Food Chem..

[5] Liu J., Kanetake S., Wu Y.H., Tam C., Cheng L.W., Land K.M., Friedman M. (2016). Antiprotozoal effects of the tomato tetrasaccharide glycoalkaloid tomatine and the aglycone tomatidine on mucosal trichomonads. J. Agric. Food Chem..

[6] Tamasi G., Pardini A., Bonechi C., Donati A., Pessina F., Marcolongo P., Gamberucci A., Leone G., Consumi M., Magnani A. (2019). Characterization of nutraceutical components in tomato pulp, skin and locular gel. Eur. Food Res. Technol..

[7] Pardini A., Consumi M., Leone G., Bonechi C., Tamasi G., Sangiorgio P., Verardi A., Rossi C., Magnani A. (2021). Effect of different post-harvest storage conditions and heat treatment on tomatine content in commercial varieties of green tomatoes. J. Food Comp. Anal..

[8] Serratì S., Porcelli L., Guida S., Ferretta A., Iacobazzi R.M., Cocco T., Maida I., Tamasi G., Rossi C., Manganelli M. (2020). Tomatine displays antitumor potential in in vitro models of metastatic melanoma. Int. J. Mol. Sci..

[9] Marcolongo P., Gamberucci A., Tamasi G., Pardini A., Bonechi C., Rossi C., Giunti R., Barone V., Borghini A., Fiorenzani P. (2020). Chemical characterisation and antihypertensive effects of locular gel and serum of Lycopersicum esculentum L. var. “Camone” tomato in Spontaneously Hypertensive Rats. Molecules.

[10] Tamasi G., Baratto M.C., Bonechi C., Byelyakova A., Pardini A., Donati A., Leone G., Consumi M., Lamponi S., Magnani A. (2019). Chemical characterization and antioxidant properties of products and by-products from *Olea europaea* L. Food Sci. Nutr..

[11] Schreiner M., Mewis I., Huyskens-Keil S., Jansen M.A.K., Zrenner R., Winkler J.B., O’Brien N., Krumbein A. (2012). UV-B-induced secondary plant metabolites-potential benefits for plant and human health. Crit. Rev. Plant. Sci..

[12] Postler T.S., Ghosh S. (2017). Understanding the holobiont: How microbial metabolites affect human health and shape the immune system. Cell Metab..

[13] Bonechi C., Lamponi S., Donati A., Tamasi G., Consumi M., Leone G., Rossi C., Magnani A. (2017). Effect of resveratrol on platelet aggregation by fibrinogen protection. Biophys. Chem..

[14] Leicach S.R., Chludil H.D. (2014). Plant secondary metabolites: Structure–activity relationships in human health prevention and treatment of common diseases. Stud. Nat. Prod. Chem..

[15] Bonechi C., Donati A., Tamasi G., Leone G., Consumi M., Rossi C., Lamponi S., Magnani A. (2018). Protective effect of quercetin and rutin encapsulated liposomes on induced oxidative stress. Biophys. Chem..

[16] Tiwari R., Rana C.S. (2015). Plant secondary metabolites: A review. Int. J. Eng. Res. Gen. Sci..

[17] Liao B., Chen X., Han J., Dan Y., Wang L., Jiao W., Song J., Chen S. (2015). Identification of commercial Ganoderma (Lingzhi) species by ITS2 sequences. Chin. Med..

[18] Bonechi C., Donati A., Tamasi G., Pardini A., Rostom H., Leone G., Lamponi S., Consumi M., Magnani A., Rossi C. (2019). Chemical characterization of liposomes containing nutraceutical compounds: Tyrosol, hydroxytyrosol and oleuropein. Biophys. Chem..

[19] Pei Y., Wu L., Zhang Q., Wang Y. (2019). Geographical traceability of cultivatedParis polyphylla var. yunnanensis using ATR-FTMIR spectroscopy with three mathematical algorithms. Anal. Methods.

[20] Carballo T., Gil M.V., Gomez X., Gonzalez-Andres F., Moran A. (2008). Characterization of different compost extracts using fourier-transform infrared spectroscopy (FTIR) and thermal analysis. Biodegradation.

[21] Durazzo A., Kiefer J., Lucarini M., Camilli E., Marconi S., Gabrielli P., Aguzzi A., Gambelli L., Lisciani S., Marletta L. (2018). Qualitative analysis of traditional italian dishes: FTIR approach. Sustainability.

[22] Bunghez I.R., Raduly M., Doncea S., Aksahin I., Ion R.M. (2011). Lycopene determination in tomatoes by different spectral techniques (UV-VIS, FTIR and HPLC). Digest J. Nanomater. Biostruct..

[23] Tan C.P., Che Man Y.B. (2002). Comparative differential scanning calorimetric analysis of vegetable oils: I. Effects of heating rate variation. Phytochem. Anal..

[24] Kotti F., Chiavaro E., Cerretani L., Barnaba C., Gargouri M., Bendini A. (2009). Chemical and thermal characterization of Tunisian extra virgin olive oil from Chetoui and Chemlali cultivarsand different geographical origin. Eur. Food Res. Technol..

[25] Tian Y., Li Y., Xu X., Jin Z. (2011). Starch retrogradation studied by thermogravimetric analysis (TGA). Carbohydr. Polym..

[26] Manara P., Vamvuka D., Sfakiotakis S., Vanderghem C., Richel A., Zabaniotou A. (2015). Mediterranean agri-food processing wastes pyrolysis after pre-treatment and recovery of precursor materials: A TGA-based kinetic modeling study. Food Res. Int..

[27] Kozukue N., Friedman M. (2003). Tomatine, chlorophyll, β-carotene and lycopene content in tomatoes during growth and maturation. J. Sci. Food Agric..

[28] Leone G., Consumi M., Franzi C., Tamasi G., Lamponi S., Donati A., Magnani A., Rossi C., Bonechi C. (2018). Development of liposomal formulations to potentiate natural lovastatin inhibitory activity towards 3-hydroxy-3-methyl-glutaryl coenzyme A (HMG-CoA) reductase. J. Drug Deliv. Sci. Technol..

[29] Leone G., Consumi M., Pepi S., Lamponi S., Bonechi C., Tamasi G., Donati A., Rossi C., Magnani A. (2017). Alginate-gelatin formulation to modify lovastatin release profile from red yeast rice for hypercholesterolemia therapy. Ther. Deliv..

[30] Khiari B., Moussaoui M., Jeguirim M. (2019). Tomato-processing by-product combustion: Thermal and kinetic analyses. Materials.

[31] Vega D., Villar M.A., Failla M.D., Vallés E.M. (1996). Thermogravimetric analysis of starch-based biodegradable blends. Polym. Bull..

[32] De Nardo T., Shiroma-Kian C., Halim Y., Francis D., Rodriguez-Saona L.E. (2009). Rapid and simultaneous determination of lycopene and β-carotene contents in tomato juice by infrared spectroscopy. J. Agric. Food Chem..

[33] Heredia-Guerrero J.A., Benítez J.J., Domínguez E., Bayer I.S., Cingolani R., Athanassiou A., Heredia A. (2014). Infrared and Raman spectroscopic features of plant cuticles: A review. Front. Plant. Sci..

[34] Yap K.Y.L., Chan S.Y., Lim C.S. (2007). Infrared-based protocol for the identification and categorization of ginseng and its products. Food Res. Int..

[35] Osborne J.W., Costello A.B. (2004). Sample size and subject to item ratio in principal components analysis. Pract. Asses. Res. Eval..

[36] USDA (1997). United States Standards for Grades of Fresh Tomatoes.

[37] Leone G., Consumi M., Lamponi S., Bonechi C., Tamasi G., Donati A., Rossi C., Magnani A. (2019). Thixotropic PVA hydrogel enclosing a hydrophilic PVP core as nucleus pulposus substitute. Mater. Sci. Eng. C.

[38] Leone G., Consumi M., Pepi S., Pardini A., Bonechi C., Tamasi G., Donati A., Lamponi S., Rossi C., Magnani A. (2020). Enriched gellan gum hydrogel as visco-supplement. Carbohydr. Polym..

[39] Garcia-Jares C.M., Médina B. (1997). Application of multivariate calibration to the simultaneous routine determination of ethanol, glycerol, fructose, glucose and total residual sugars in botrytized-grape sweet wines by means of near-infrared reflectance spectroscopy. Fresenius J. Anal. Chem..

[40] Oliveri P. (2017). Class-modelling in food analytical chemistry: Development, sampling, optimisation and validation issues. A tutorial. Anal. Chim.Acta.

[41] Shah N., Cynkar W., Smith P., Cozzolino D. (2010). Use of attenuated total reflectance midinfrared for rapid and real-time analysis of compositional parameters in commercial white grape juice. J. Agric. Food Chem..

[42] Li B., Martin E., Morris J. (2001). Latent variable selection in partial least squares modelling. IFAC Proc. Vol..

[43] Croce R., Malegori C., Oliveri P., Medici I., Cavaglioni A., Rossi C. (2020). Prediction of quality parameters in straw wine by means of FT-IR spectroscopy combined with multivariate data processing. Food Chem..

[44] Silalahi D.D., Midi H., Arasan J., Mustafa M.S., Caliman J.P. (2018). Robust generalized multiplicative scatter correction algorithm on pretreatment of near infrared spectral data. Vib. Spectrosc..

[45] Barnes R.J., Dhanoa M.S., Lister S.J. (1989). Standard normal variate transformation and de-trending of near-infrared diffuse reflectance spectra. Appl. Spectrosc..

[46] Savitzky A., Golay M.J.E. (1964). Smoothing and differentiation of data by simplified least squares procedures. Anal. Chem..

[47] Wold S., Sjöström M., Eriksson L. (2001). PLS-regression: A basic tool of chemometrics. Chemom. Intell. Lab. Syst..

[48] Forina M., Latenri S., Cerrato Oliveros M.C., Pizarro Millan C. (2004). Selection of useful predictors in multivariate calibration. Anal. Bioanal. Chem..

